# Lived experience of driving in individuals with functional neurological disorder

**DOI:** 10.1002/brb3.3652

**Published:** 2024-08-21

**Authors:** Tjerk J. Lagrand, Iris van der Hoeven, Atiyeh Vaezipour, David D. G. Palmer, Andrew Hill, Mark S. Horswill, Alexander C. Lehn

**Affiliations:** ^1^ Department of Neurology Alrijne Ziekenhuis Leiderdorp Zuid‐Holland The Netherlands; ^2^ RECOVER Injury Research Centre The University of Queensland Brisbane Australia; ^3^ Department of Neurology Princess Alexandra Hospital Woolloongabba Queensland Australia; ^4^ Minerals Industry Safety and Health Centre, Sustainable Minerals Institute The University of Queensland Brisbane Australia; ^5^ School of Psychology The University of Queensland Brisbane Australia; ^6^ Faculty of Health, School of Biomedical Sciences Queensland University of Technology Brisbane Australia

**Keywords:** driving, driving safety, functional neurological disorder, lived experience, qualitative study

## Abstract

**Background:**

Functional neurological disorder (FND) is a common neurological diagnosis that encapsulates a range of incapacitating clinical presentations. These include functional seizures, movement disorders, and sensory disturbances. Safe driving requires both cognitive skills and physical abilities, which may be impacted by FND symptoms. The primary objective of this study was to gain deeper insights into the challenges faced by people with FND when driving.

**Methods:**

A qualitative study and interpretative phenomenological analysis were conducted. Individuals experiencing functional seizures and/or movement disorders completed both questionnaires and semi‐structured interviews about FND symptoms, driving behavior, and crashes.

**Results:**

A total of 26 patients with FND participated in this study. Based on the interviews, four key themes were identified: (1) driving difficulties experienced by individuals with FND; (2) strategies utilized by people with FND to overcome difficulties experienced while driving; (3) barriers preventing driving challenges being addressed in this population; and (4) crashes and perceived dangerous driving events experienced by individuals with FND. All participants reported that driving a car provoked FND symptoms and this affected their driving ability. FND sufferers reported using a number of strategies such as limiting how far they drive and relying on advanced driver assistance system features to help manage their associated symptoms, such as fatigue and/or pain. Several participants reported crashes and perceived dangerous driving events since developing FND.

**Conclusion:**

Individuals experiencing FND often employ self‐regulation techniques, yet the extent to which these methods enhance driving safety remains uncertain. The variable nature of the disorder makes judging an individual's driving risk particularly difficult. The themes emerging from the interviews highlighted the need for further empirical research to inform guidelines and best practice when determining the impact of FND on an individual's driving safety .

## Graphical abstract

This qualitative study on individuals with FND showed that functional symptoms affect driving ability. FND sufferers report the use of various strategies to help manage their symptoms but several study participants reported crashes since developing FND.

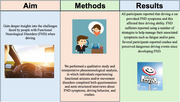

## 1 INTRODUCTION

Driving is an important part of life for many people. It is associated with a greater degree of autonomy, improved physical and mental health, and higher levels of social participation (Carr et al., 2019). However, unsafe driving involves an increased risk of crashing, potentially causing damage, injury, or even death. Road traffic crashes are a leading cause of injury and death, with 1.19 million victims globally each year (World Health Organisation, 2022).

As many medical and neurological conditions can impair a person's ability to drive, the question “Is this patient safe to drive?” remains a difficult topic in neurology outpatient clinics. A study suggests that deficits in visual, cognitive, and motor skills among individuals with epilepsy have a notable impact on their ability to drive safely (Classen et al. (2012). Common errors include difficulties in controlling speed, insufficient visual scanning, and failure to maintain proper lane positioning. Given the similarities between functional seizures and epileptic seizures, it is reasonable to argue that both conditions affect safe driving in similar ways. Additionally, research on patients with Parkinson's disease revealed challenges in vehicle control, such as variability in lane positioning and difficulties in stopping (Stolwyk et al., 2005). It was observed that those who struggled more with adapting to changes in their environment relied more on external cues, such as traffic signs, rather than internal cues, like memory, to guide their driving behavior. Given the resemblance of symptoms between functional movement disorders and Parkinson's disease, including tremors and cognitive issues, it is plausible to suggest that they also impact driving abilities similarly. Patients often resist recommendations to cease driving, although many do self‐regulate their behavior by not driving during rush hours, on highways, or at night (Feng et al., 2020). For some neurological conditions, such as stroke and epilepsy, national and international driving guidelines and recommendations already exist (Devlin et al., 2012; Rabadi et al., 2010). However, appropriate guidelines are lacking for other disorders.

Functional neurological disorder (FND) covers a variety of clinical presentations, in which the primary pathophysiologic mechanisms are alterations in functioning of brain networks rather than structural abnormalities in the brain (American Psychiatric Association, 2013). Although long recognized, this disorder was largely neglected by healthcare professionals, and was given various diagnostic labels, including conversion, psychogenic, and dissociative disorder. This psychocentric viewpoint often led to management issues by neurologists, resulting in inadequate attention to critical issues such as driving impairments. However, contemporary perspectives have shifted significantly, recognizing FND as a legitimate neurological condition (Perez et al., 2021). The two most common subtypes are functional seizures (also known as dissociative attacks) and functional movement disorders (FMD). Individuals with functional seizures experience periods of altered or absent consciousness and/or abnormal movements resembling epileptic seizures or syncope. FMDs can manifest as any type of abnormal movement, such as tremor, dystonia, myoclonus, or weakness (Hallett et al., 2022). In addition, fatigue, chronic pain, and cognitive symptoms often referred to as “brain fog” are common in all subtypes of FND (Cock & Edwards, 2018).

Patients with FND might be expected to experience a range of driving‐related difficulties either as a direct result of FND (e.g., motor or sensory symptoms or cognitive impairment) or as a result of comorbid psychiatric conditions that can accompany FND, such as anxiety and depression (Pun et al., 2020). It could also be hypothesized that FND patients' attention could be diverted away from their symptoms as some patients report fewer problems when driving.

Most countries have clear guidelines regarding driving restrictions for a variety of health conditions, such as epileptic seizures. Only some countries (like the UK and Australia) (GOV.UK. (n. d.); Austroads, 2022b) have specific regulations for patients suffering from functional seizures and no clear guidelines exist for other functional neurological symptoms. One reason for this is likely that the impact on driving safety by these conditions is far less clear. This lack of clarity about driving regulations has a major impact on patients and health professionals: For patients restrictions on driving are a major concern (Xu et al., 2015), and discussions about driving restrictions are challenging for doctors, particularly when guidelines are not clear (Farooq et al., 2018). While there has been growing interest in the impact of FND on driving ability (Lagrand et al., 2023), much of the available literature consists of expert opinion and case studies, but the current literature does not capture the experiences of patients with FND and their challenges with driving (Chang et al., 2023; Kanaan et al., 2022; Stone et al., 2005). Patient experiences and perspectives can be a valuable source of information for identifying areas of need and informing interventions.

This study aimed to enhance our understanding of the driving experience of adults with FND. The goal of this research is to inform recommendations and interventions, including public education, to ensure the fitness to drive of people with FND. A qualitative research design and interpretative phenomenological analysis (IPA) were conducted to provide insight into driving challenges and strategies to overcome these by individuals with FND.

## 2 METHODS

### 2.1 Participants

Prospective and sequential recruitment was conducted among patients diagnosed with functional seizures and/or functional movement disorders between January and July 2022 from two institutions in Southeast Queensland, Australia: The Princess Alexandra Hospital, a quaternary level teaching hospital, and the Brisbane Clinical Neuroscience Centre, a private practice with a special interest in FND. The diagnosis was established by a neurologist with expertise in movement disorders and FND (AL).

Eligibility criteria were that participants: (1) had experienced functional seizures or functional motor symptoms over the last year, with symptoms causing distress or impairment in social, occupational, or other important areas of functioning (definition according to DSM 5) (Rabadi et al., 2010); (2) were over the age of 18 years; (3) held an open Australian driver license; and (4) had not been diagnosed with a neurological condition other than FND that could influence driving behavior, such as epilepsy or Parkinson's disease. Eligible participants were asked to complete a demographic and FND questionnaire (see section 2.3 for details) and were invited to take part in a semi‐structured phone interview with a member of the research team (T. L. or D. P.).

### 2.2 Procedure

This research adhered to the National Statement on Ethical Conduct in Human Research. Participants were informed that their participation was entirely voluntary, they were free to withdraw at any time, and they could choose not to answer any question(s). Participants were assured that their responses would be recorded anonymously, and questions about on‐road behavior did not seek to elicit details (e.g., times or locations) that could be used to make specific instances of disclosed behaviors identifiable (to minimize any potential legal harm). They were told that no identifying information would ever be made public or disclosed to authorities unless required by law. Participants were also advised that, if they had experienced a crash and felt uncomfortable recalling their experience, they should consider not participating in the study. They were also told that counseling services were available to them if they became upset or distressed because of their participation in the study. If participants admitted to engaging in any concerning driving behaviors, then researchers encouraged them to discuss this with their clinician.

Ethical clearance was granted by the Metro North Health Human Research Ethics Committee. All data were collected and stored anonymously in accordance with the Data Protection Act (1998).

Participants were interviewed over the phone for 30–60 min. A semi‐structured interview guide was developed by Tjerk J. Lagrand and Atiyeh Vaezipour and reviewed by other members of the research team (D. P. and A. L.). The interview covered various topics related to driving experiences, self‐regulation strategies, and barriers to addressing driving challenges with healthcare providers. The interview guide can be found in Supporting information Appendix.

Each participant was interviewed by a neurologist with specialty interest in FND (T. L. or D. P.), who did not have a prior relationship with them. All interviews were audiorecorded and transcribed verbatim. The final number of participants was determined by data saturation, where additional interviews provided limited new information.

### 2.3 Demographic and FND questionnaire

Demographic information was collected on participants’ gender, age, highest educational attainment, employment status, and driving experience. FND characteristics recorded (based on the investigation by a neurologist specialized in FND) were subtype, duration of symptoms, diagnostic delay, and last attack or flare of symptoms. In addition, participants were asked to self‐report the occurrence of motor vehicle crashes before and since the onset of their symptoms, and if they believed that their FND contributed to their motor vehicle crash. The response options were “yes” and “no,” but respondents were able to provide additional elaboration if desired. A motor vehicle crash was defined as any incident involving a vehicle that resulted in personal injury or damage to a vehicle or other property.

### 2.4 Data analysis and trustworthiness

Transcripts were analyzed by two researchers with experience in qualitative data analysis (T. L. and I. H.). An IPA was used to guide the qualitative analysis. The process involved familiarization with interview transcripts, independently identifying meaningful text, and formulating common themes from exploratory notes. A coding system was developed from discussions between Tjerk J. Lagrand and Iris van der Hoeven regarding various topics from each transcript. Following analysis of individual transcripts, a cross‐case analysis was conducted, identifying shared themes across all transcripts.

The trustworthiness of the coding was established using reflexive discussions between the researchers (T. L. and I. H.) throughout the analysis. Additionally, three other researchers (D. P., A. V., and A. L.) reviewed the themes identified. Discrepancies were discussed and guided the thematic development, resulting in a precise set of meanings for participant comments.

## 3 RESULTS

A total of 26 individuals participated in the study, consisting of 19 females and 7 males aged 24–75 years (mean = 46.1, SD = 12.9). Participants had a mean of 28.9 years of driving experience (SD = 13.2, range 6–58 years). Participants reported driving an estimated 4995 km (SD = 9736, range 150–30,000 km) per year and 6.9 h (SD = 6.9, range 0–21 h) on average per week. Functional seizures (69%) were more common than functional movement disorders (FMD, 31%). There was a wide range of duration and symptoms in both groups, although 15 patients (58%) reported an attack within the last 2 weeks. De‐identified details of the individual participants’ demographics and FND characteristics are included in Table 1.

**TABLE 1:** Demographics and characteristics.

	Functional seizures (*n *= 18)	Functional movement disorders (*n* = 8)	Combined (*n *= 26)
Sex (*n*, % female)	14 (78%)	5 (63%)	19 (73%)
Age (mean + SD, min–max)	47 ± 10.3 (24–68)	48 ± 17.2 (24–75)	47 ± 12.9 (24–75)
Duration, months (median, range)	50 (6–483)	55 (17–82)	51 (6–483)
Diagnosis, months (median, range)	23 (5–184)	28 (8–60)	25 (5–184)
Last seizure/flare, days (median, range)	12 (1–214)	6 (1–275)	8 (1–275)
Highest education (*n*, %) High school Undergraduate Trade qualification Postgraduate	2 (25) 5 (63) 0 1 (13)	5 (28) 5 (28) 4 (22) 4 (22)	7 (27) 10 (38) 4 (15) 5 (19)
Employment status (*n*, %) —Unemployed —Part time —Full time	3 (38) 1 (13) 4 (50)	12 (67) 3 (17) 3 (17)	15 (58) 4 (15) 7 (27)
Self‐reported crashes —Before symptoms —After symptoms —Related to FND	12 3 1	21 3 3	33 6 4

Abbreviation: FND, functional neurological disorder.

The following analysis presents the viewpoints of individuals with FND on four key themes: (1) driving difficulties experienced by individuals with FND, (2) strategies utilized by people with FND to overcome difficulties experienced while driving, (3) the barriers to adequately addressing driving challenges for this population, and (4) crashes and potentially dangerous driving among individuals with FND.

### 3.1 Driving difficulties experienced by individuals with FND

Table 2 presents the number of patients who have particular driving difficulties. All participants stated that the extended and repetitive movements involved in operating a motor vehicle provoked FND symptoms and/or affected their driving ability. Twenty‐four of the participants (92%) reported that the act of driving could enhance functional seizures or flares of motor symptoms. For example,

**TABLE 2:** Key theme 1: Driving difficulties experienced by individuals with functional neurological disorder (FND).

Subthemes	Functional seizures (*n* = 18)	Functional movement disorders (*n* = 8)	Combined (*n* = 26)
Driving can cause FND attacks or flares of motor symptoms	16	8	24
Driving can flare up fatigue or exhaustion	13	7	20
Physical and cognitive difficulties due to FND lead to slower reaction time or other driving difficulties	12	4	16
Driving can negatively impact on the mood (i.e., gets anxious about having to react suddenly)	2	6	8
Driving can flare up associated pain	1	6	7
Difficulties when driving close to bright lights or with loud noises	1	6	7
Difficulties driving at night, in rain or heavy fog	0	7	7

*That has happened on longer journeys when you've felt fine when you departed but then, halfway through, you start to get some symptoms. That's happened a handful of times. First, I tend to go very quiet, and my speaking gets difficult. After that, the weakness starts. (P17)*

Participants also noted that driving could cause a rise of their associated features in FND, including fatigue and pain. With respect to fatigue, 20 participants (77%) mentioned that driving resulted in tiredness and exhaustion.

*We were on holiday and then suddenly the fatigue really hit me badly. I've been driving for 40 minutes, I guess, even though it was an open road, no traffic, cruise control all that, and had my partner sitting beside me talking to me. It just wiped me out. It just absolutely wiped me out. (P14)*

*I am a bit afraid to tell, but I fainted previously from exhaustion and hit my head on the steering wheel. (P8)*

Sixteen participants (62%) perceived that FND impacted their perceptual‐cognitive abilities, which had an impact on their attention and reaction time.

*Before my FND symptoms started, the way I drive was, how would you say it, assertive? It was quite dynamic because that's how I got taught to drive when I was a police officer… Nowadays, I must be more careful because the brain fog will come in and my responses are a lot slower than they used to be. (P11)*

*I can't focus. I can't think. I can't concentrate. (P20)*

*I do struggle with my reaction time. Let's say someone pulls out in front of you. Sometimes I delay beeping the horn at them when you try and alert them. My husband's been telling me a couple weeks ago I'm braking too late to stop at the lights. (P21)*

Eight participants (31%) reported a negative impact on their mood, either decreased ability to cope with distress, increased anxiety because of experiencing functional symptoms, or both. For example,

*I ended up just crying and shaking because I was so frightened. (P24)*

*I don't have any difficulties with reversing or merging, but I sometimes feel anxious about it, particularly if there are aggressive or dangerous drivers around me. I was a little bit anxious about that before FND. I think my FND has just made it stronger. (P23)*

Seven patients (27%) mentioned an increase in associated pain. For example,

*I've been sitting there in my car, and then after like 10–15 minutes the really painful pins and needles happen. (P1)*

Seven participants (27%) indicated that their FND affects their driving in challenging sensory situations, for example bright lights or loud sounds.

*If I'm driving and someone has fluoro high beams or bright headlights, even sometimes just general light as well, that can flare up my symptoms. There's a couple of signs around here at roundabouts, like advertisements that are particularly blinding. (P1)*

*The more noise, the worse my perception is going to become. (P21)*

Finally, adverse weather conditions were also problematic for seven patients (27%).

*I don't drive at night if there's not enough light. I know that my FND can easily be triggered. Raining can trigger it too heavily. (P20)*

### 3.2 Strategies utilized by people with FND to overcome difficulties experienced while driving

Table 3 presents different strategies utilized by patients to overcome their driving difficulties. All participants reported some degree of subjective impairment in their driving ability due to FND. However, participants employed various strategies to handle these challenges and manage driving responsibilities and FND‐related issues safely. These strategies can be categorized as a type of self‐regulation of driving behavior. All but one patient described specific strategies they used to help themselves drive more safely and comfortably despite their symptoms.

**TABLE 3:** Key theme 2: Strategies utilized by people with functional neurological disorder (FND) to overcome difficulties experienced while driving.

Subthemes	Functional seizures (*n* = 18)	Functional movement disorders (*n* = 8)	Combined (*n* = 26)
Avoids driving or asks others to drive during FND flare‐ups or when feeling unwell	18	7	25
Drives automatic car most of the time	16	7	23
Drives less frequently/shorter distances	13	6	19
Uses pacing strategies to prevent flare‐ups of FND while driving	11	5	16
Avoids driving in high volume traffic/peak hours/unfamiliar roads/distraction	11	5	16
Uses driver assistance systems, that is, cruise control to adhere with speed limits, blind spot detectors, and navigation	11	5	16
Relaxation strategies and/or slower driving to assist with the cognitive aspect of driving	13	2	15
Takes more time with preparing route planning	6	5	11
Tries to be more attentive and vigilant	7	4	11
Listens to (loud) music and sings/asks someone to talk to them to distract oneself	4	0	4
Lock it out of the limbs that the person is driving with, holding the steering wheel tighter	2	1	3
Asks drivers with more experience to assess the driving	3	0	3
Uses a heated steering wheel to prevent holding the wheel too tight	1	0	1

The most common strategy used by participants was to request someone else to drive (96%).

*When I don't have to drive, I don't drive. My wife drives. (P14)*

*My son and my mum know if I ring and say, ‘I need you to drive,’ they know automatically what's going on. (P20)*

Three participants (12%) said that they avoid driving alone without an experienced driver sitting in the passenger seat to assess the driving:

*I've asked my husband to monitor my driving to see whether he thinks I'm still safe…I get him to observe my driving as a secondary form of information data. (P23)*

Participants also discussed varying pacing strategies, for example, breaking up the drive, reducing the frequency of trips, decreasing the distance of the drive, or taking an extra rest before a trip. Nineteen participants (73%) reported reducing the frequency and/or length of driving trips.

*I don't drive far, like I really have to push myself to drive 5 or 10 minutes. (P8)*

*Before my FND, I could drive twelve hours straight, in one stretch, no problem… now, it becomes a problem when I'm driving for over two hours. (P11)*

Other strategies to overcome FND‐related difficulties involved planning the journey to avoid driving in certain contexts, such as high‐volume traffic or unfamiliar roads.

*I go the easy way, whether it be staying on the main road until I have to get off, where usually I would go through the city. I avoid the city now. (P4)*

*I don't drive at night, hardly at all anymore. (P7)*

*For me, it's like sensory overload with lights and sounds. Especially if I'm at a stoplight, then there's cars going past, and I have to close my eyes just so they don't trigger me. (P22)*

Fourteen participants (62%) used advanced driver assistance systems (ADAS) or other vehicle features. Most of them found these devices helpful, for example, regarding cruise control.

*It helps because when I am driving with my foot at an angle, it causes so much aching that it flares up my FND. (P1)*

*I use cruise control which I do find probably a little bit more useful if my symptoms are happening while I'm driving. These systems improve my control over the car, when I feel it bit on edge (P17)*

*I always use cruise control, mainly because I don't need to concern myself. (P25)*

Others discussed the use of multiple ADAS features at the same time.

*Cruise control, lane safety, lane keeping assist and lane following assist, the blind spot collision avoidance, the stability control and forward collision avoidance and the driver attention warning, they're generally always on in the vehicle. (P16)*

*I have all of these things for two reasons. One is to reduce my anxiety levels, which then helps with my FND symptoms. And the other is to reduce cognitive load and sensory input and not be overwhelmed. (P24)*

Interestingly, two participants (8%) had the opposite opinion about ADAS.

*I find all those bells and whistles distracting. (P15)*

*I don't think they're helpful. I think that it's better to stay vigilant. (P9)*

Vehicle systems were sometimes used for pain reduction or to control involuntary movements. For example,

*I use a seat warmer even though we're in Australia because it reduces the pain in my back. (P18)*.

*I do use a heated steering wheel so that I don't grip the steering wheel to death. (P23)*

Fifteen (58%) participants discussed using relaxation strategies and/or slower driving to assist with the cognitive aspect of driving. For example,

*I must do the breathing exercises while I'm driving to stay relaxed. (P10)*

*I always keep an anti‐stress ball in the car, and it distracts by squeezing or throwing it. I'm only using it at a red light for example. (P19)*

*I just drive like a granny now. Very slowly, always in the left lane. (P22)*

Eleven participants (42%) tried to be more attentive and vigilant while driving.

*I am extra vigilant on the road. Not quite paranoid, but just conscious that you've got something that most people don't have with the potential that it could affect your driving. (P17)*

Finally, seven participants (27%) shared how performing a secondary task for distraction could help to reduce FND symptoms while driving:

*I either play music really loud or sing the songs myself to keep engaged. (P12)*

*I still have regular jerking episodes, but I can lock those out by holding the steering wheel real tight. (P6)*

### 3.3 Barriers to adequately addressing driving challenges for this population

Table 4 presents the obstacles patients perceive to addressing the barriers they face while driving. Among the participants, seventeen (65%) stated that health professionals did not bring up the subject of driving during their evaluations or treatments.

**TABLE 4:** Key theme 3: Barriers to adequately addressing driving for individuals with functional neurological disorder (FND).

Subthemes	Functional seizures (*n* = 18)	Functional movement disorders (*n* = 8)	Combined (*n* = 26)
Health professionals inadequately address driving	11	6	17
Health care professional cannot take individual circumstances under consideration due to the current guidelines	10	4	14
Has not discussed driving difficulties with health professionals, fear of limiting driving	2	2	4

*Before this study, no health professional ever questioned my driving. That's what surprised me, and to be honest it scares me because I know people on my own FND Facebook group that drive, and they tell me about some of the issues they have. (P14)*

Additionally, 14 participants (54%) mentioned that one of the reasons that health professionals inadequately address driving difficulties could be the variety of symptoms.

*There's a whole menagerie of FND symptoms, some of which would have no effect on driving. It's not going to be a one size fits all approach. (P12)*

*It's all on an individual basis. My daughter, who is 27 and has FND too, she got it probably 10 times worse than I have it. (P13)*

Some participants discussed ways to overcome these barriers.

*I'd also like the opportunity to drive with an allied health professional to better discuss strategies to help with driving, or to have a driving assessment to better identify potential issues” and, “It's very much a recommendation that would have to be put forward to, maybe a bit like epilepsy … you've got to be FND symptom‐free for a certain period. (P14)*

For many Australians, driving is the primary means of transportation. As a result, three participants (12%) reported finding discussion of driving behavior and safety with a health professional confronting.

*I think the big issue is that as soon as a healthy person mentioned driving, I thought, that's a nightmare, the thought of having my life removed. I'm sure I changed some answers to those questions because I was fully aware of where that may lead. (P17)*

*I am not bringing these things up. I don't want to lose my license, because I need my license, because I believe it's my independence, it is who I am. I'm a teacher, so if that went, I'm gone, and I don't want that to go. I'm just very cautious of what I say. (P26)*

### 3.4 Crashes and potentially dangerous driving among individuals with FND

Participants were asked whether they had been involved in any motor vehicle crashes both ever and specifically after the onset of their FND symptoms. People who had crashes since developing FND were asked whether they believed their FND played a role in the crash. Twenty‐one participants (81%) mentioned at least one crash in the past, of which five people (19%) had had a crash after the onset of their symptoms.

One participant (4%) reported that a car crash was the precipitating factor for a functional attack.

*I was on a highway and lost control of the car. The car rolled over and once they got me out and took me into the ambulance, I had five seizures, and I never had those before…In hospital, they were doing a lot of tests on me and then I was diagnosed with FND. (P2)*

Three (12%) participants reported incidents which may have been related to FND. For example,

*I was exhausted and wanted to go home. I was leaving the car parking lot and a lady didn't give way to me and drove straight into my door. I had forgotten to put my seatbelt on, so I did a forward backwards, side to side, forward, backwards with my head and lost consciousness for a couple of minutes…this crash could have been related to FND in the sense that I was extremely tired and didn't remember to put my seat belt on. That's almost always automatically what I do. I was shocked when I realized I didn't have my seat belt on. I even asked the lady if she had taken my seatbelt off, and she said she hadn't. (P9)*

*I had a dissociative attack in the car two weeks ago. I was falling asleep while I was driving. So, when I say ‘falling asleep’, I was starting to get unconscious. I had my friend follow me home. (P20)*

In one participant, an attack of functional leg paralysis resulted in a potentially dangerous driving incident:

*I was driving myself and I think it was with three work colleagues back from a work event. I was approaching the lights and the light was orange and I was trying to brake, and my leg wasn't responding. I had no choice but to go through the orange light. Thankfully, it was safe to do so. (P9)*

A small number of participants reported driving with significant symptoms in circumstances that most people would deem excessively risky.

*I wanted someone to drive the car for me, but it was a manual, and none of the other passengers could drive manual. I just had to do my right hand on my right leg, and every time I needed to brake, I would just slap it, or I would lift it and we got back. (P9)*

*I get these tight, very painful spasms in my fingers and often have to use one hand to hold the steering wheel then rotate with the other hand. So, it's very rare that I've got both hands on the steering wheel at one time. (P24)*

*I've driven with my legs shaking. I've driven holding one hand down on the accelerator because I haven't had strength in my legs to hold them. (P21)*

Finally, to the question of whether they thought they could confidentially assess if they were safe enough to drive, negative answers were given by five participants (19%). Illustrative examples of their comments are:

*I believe initially it's trial and error. (P22)*

*I could definitely assess myself, but whether I would be judgmental of myself? No, if I want to drive, I will drive, regardless of how I feel. And that's one of the issues I have. (P14)*

*I have to say I don't know but that's with hindsight. At the time, I would have told you categorically I know when I can, and I know when I can't. But as I think of that now, I have to say, I really don't know. (P21)*.

## 4 DISCUSSION

This qualitative study explored how FND affects driving, as seen from the experiences of individuals living with the condition. All participants felt that their functional symptoms affected their ability to drive a vehicle safely and most reported flares of functional symptoms when driving (particularly with prolonged driving). Commonly reported issues were flares of pain and difficulty with attention and impaired reaction time. Another concern that was raised frequently was difficulty with managing stressful driving situations or situations causing sensory overload, such as driving at night or driving in heavy traffic. A number of participants reported experiencing highly concerning driving incidents caused directly by their FND symptoms, such as unexpected functional leg paralysis impeding braking at a safety‐critical moment. All but one participant reported using specific strategies to overcome driving difficulties and improve driving safety. These strategies are similar to those used by other patient groups with issues that could impair the ability to drive, such as those with Parkinson's and chronic pain, who have also been found to adopt strategies such as driving avoidance and self‐restriction, journey planning, and pacing strategies such as having intermittent breaks from driving (Brock et al., 2021; Crizzle et al., 2013; Hassan et al., 2017; Vaezipour et al., 2022).

Participants in this study expressed concern about losing their driver's license and fearing the resulting loss of independence. The majority of participants reported that the driving safety implications of their FND symptoms were not a conversation topic with their healthcare providers. There could be several reasons for this, such as (1) time–pressure during patient reviews, (2) concerns about damaging the patient–doctor relationship, and (3) that health professionals often share limited knowledge about driving guidelines and their implications. It could also be that, among health professionals, there is still a perception that patients with FND can control their symptoms sufficiently if needed, allowing them to drive safely. The current data suggest that this might not be true for all patients, where some symptoms, like functional leg paralysis, can potentially occur without warning in a way likely to compromise safety when driving.

Study findings indicate that addressing driving safety in patients with FND is both challenging and complex. The characteristics of the disorder, with its episodic nature and often dramatic fluctuations of symptoms, makes judging driving risk particularly difficult. People suffering from FND commonly use self‐regulation strategies, but it is not clear how effective these strategies are in improving driving safety. Due to the variability seen with this disorder, it would be difficult to develop generic driving advice that could reasonably apply to the breadth of symptoms and symptom‐triggers that people with FND experience. In 2020, the International League Against Epilepsy (ILAE) published recommendations for patients with functional seizures based on a systematic literature review and expert opinion, and concluded that decisions about whether a patient can continue to drive should be made at an individual level (Asadi‐Pooya et al., 2020). More work needs to be done to develop strategies for assessing fitness to drive in this patient group, as well as in other FND phenotypes for which recommendations are lacking, for example, functional movement disorders. A driver‐centered approach to determine whether patients can still drive a motor vehicle safely might be warranted. By doing that, features such as symptom severity, compliance to (physio)therapy, prior crashes, or a driver's history of traffic violations associated with their FND symptoms could be taken into account. Note that this type of driver‐centered approach is in place for other impairments, such as epilepsy, where driving is allowed if deemed safe for a particular individual (which might include assessment outcomes, compliance to recommendations, self‐regulation, and management of risk). (Austroads, 2022a)

Given that the present study relied on participants’ subjective self‐insights, further research with a larger sample would be of value. Employing objective driving data and a control group without FND would strengthen the findings and allow for causal inferences. Furthermore, the impact of FND on driving risk should be examined separately for different subgroups based on symptom type, as well as consistency and severity of symptoms, to tackle the issue of group heterogeneity. This type of approach would be expected to yield more actionable findings with respect to the clinical management of individuals with FND, potentially reducing their overall crash risk without imposing inappropriate restrictions on their driving autonomy.

## AUTHOR CONTRIBUTIONS

**Tjerk J. Lagrand**: Conceptualization; investigation; writing—original draft; methodology; writing—review and editing; formal analysis; data curation. **Iris van der Hoeven**: Writing—original draft; writing—review and editing; formal analysis; data curation. **Atiyeh Vaezipour**: Conceptualization; investigation; methodology; writing—review and editing; formal analysis. **David D. G. Palmer**: Formal analysis; conceptualization; investigation; writing—review and editing; data curation. **Andrew Hill**: Writing—review and editing; supervision; methodology; conceptualization. **Mark S. Horswill**: Writing—review and editing; supervision; conceptualization; methodology. **Alexander C. Lehn**: Conceptualization; investigation; writing—review and editing; methodology; supervision.

## FUNDING INFORMATION

This research received no specific grant from any funding agency in the public, commercial, or not‐for‐profit sectors.

## CONFLICT OF INTEREST STATEMENT

The authors declare no conflicts of interest.

### PEER REVIEW

The peer review history for this article is available at https://publons.com/publon/10.1002/brb3.3652

## Supporting information

Supporting Information

## Data Availability

The data that support the findings of this study are available from the corresponding author upon reasonable request.
