# Supplementary material for: Lived experience of driving in individuals with functional neurological disorder
Source: Brain Behav. 2024 Aug 21;14(8):e3652. doi: 10.1002/brb3.3652 (PMC11338837; doi:10.1002/brb3.3652)
Supplement: Supplementary file 1 — Supporting Information [file BRB3-14-e3652-s001.docx]

QUESTIONNAIRE

Individuals with FND Interview Guide Questions

Participant Code to keep the data private and anonymous and use non-identifiable codes to link the data through the study. Participants to create their own code:

- *First three letters of your mother’s maiden name (3) Month of your birthday (2) Postcode (4)*

Interview guide questions

*Please remember it is completely voluntary to answer these questions. If at any time you would prefer not to answer the question, just say so and we will move on to the next question. Also, be assured that your answers to the questions today will have absolutely no impact on your licensing or ability to drive.*

1. **What types of transportation do you regularly or occasionally use?**

*Prompts: Drive yourself, have someone drive you, use public/alternative transport.*

1. **What is your preferred mode of transport and why? Can you describe any alternative transportation options that you would potentially use instead of driving? (advantages/disadvantages of these options)**

*Prompts: ease, affordability, independence.*

1. **When thinking about your FND symptoms, do you think there have been any changes in how you drive now, compared to how you used to drive before your FND started? If so, what changes have you seen?**
2. **Does your FND impact your ability to perform the following tasks involved with driving?**
3. Preparation of driving and consideration of routes. **If yes, how and what strategies do you adopt to overcome these difficulties?**

*Prompt: examples of tasks in preparation to drive include getting into the car, adjusting your mirrors/chair, fastening your seat belt, route planning, driving at night/traffic*

1. Performing driving maneuvers**: If so, how and what strategies do you adopt to overcome these difficulties?**

*Prompt:* *examples of tasks include reversing, merging with traffic, turning, streeing,*

1. Vehicle control**: If so, how and what strategies do you adopt to overcome these difficulties?**

*Prompt: examples of tasks include steering control, accelerating, and braking,speed control, reaction time*

1. When was your last dissociative attack/flare of your functional neurological disorder (FND) and can you describe what happened?

*Prompts:* *triggers, warning signs, loss of consciousness, problems with movement, ED presentation, etc*

1. **Have you had any dissociative attacks/flares of your functional neurological disorder (FND) or warning signs while driving, and if so, how did you deal with it?**

*Prompts:* *Could you recognize warning signs? Could you hold back the buildup? Could you pull aside? Could you continue driving despite? Could you use your strategies mentioned in the previous question?*

1. **Do you think your FND reduces your capabilities to deal with certain complex driving situations? If so, how and what strategies do you adopt to overcome these difficulties?**

*Prompts for tasks*: *Did you need to* *avoid driving in certain traffic conditions e.g. congested/high traffic, at night, heavy rain, on an unfamiliar route, driving alone, etc. Can you think of any other situations?*

*Prompt for strategies:* limit driving at night, limit driving in rush-hour traffic, limit driving alone.

1. **Does driving negatively impact you in any other way e.g., your ability to do other activities after driving? If so, in what way?**

*Prompts:* *Is driving maybe a trigger for flares of FND/symptoms? Does it impact housework, sitting or standing, impact on your relationships with others, emotional well-being.*

1. **When thinking about your FND, can you confidently assess when you are and are not safe to drive? Why/why not?**

*Prompts: For example, Fatigue, mental effort, attention, memory.*

1. **Have you ever found yourself in a situation where you felt you were unsafe to drive because of your condition? If so, can you describe the situation and what action you took?**

*Prompts:* *For example, Near misses, unpredictable traffic flow, pressure from other drivers.*

1. **Do you currently use any in-car system (e.g., cruise control), and if so, what driver assistance systems do you think would make driving with FND safer?**

*Prompts:* *For example, Cruise control, blind spot monitor, etc.*

1. **Has a family member or friend ever suggested that you should limit your driving or stop driving due to concerns associated with your FND?**

*Prompts:* *For example, Partner, children, friends, work colleagues, parents, siblings.*

- 1. **Who and what were their concerns?**
  2. **What, if any, actions did you take in response to their concerns?**

1. **Has a health care professional ever asked you about your driving?**

*Simple yes/no response.*

1. **If yes, have any of these health care professionals ever advised or suggested that you should limit your driving or stop driving due to concerns associated with your FND?**

*Prompts:* *GP, neurologist, occupational therapist, other specialist.*

- 1. **What were their concerns?**
  2. **Did they work with you to develop strategies for overcoming difficulties and to ensure your safety while driving?**

*Prompts:* *For example suggested other forms of transportation, suggested specific instances in which to limit your driving (e.g., heavy traffic, at night).*

- 1. **What, if any, actions did you take in response to their concerns?**

*Prompts: used public transport instead, asked friend/family to drive*

1. **Do you have any suggestions for how health professionals could better address assessing driving ability for individuals with FND and assisting them with continuing to drive safely?**

*Prompts:* *For example,*  *vehicle adaptations, educational materials.*

1. **Have you been involved in a traffic crash, where you were the driver, before and since developing FND?**
   1. **Can you please describe what happened?**

*Prompts: Number before FND diagnosis, number since FND diagnosis + description of accident/crash*

- 1. **What factors do you believe contributed to this crash? Were you at fault?**

*Prompts:* *Do you think your FND condition could have been contributed to this crash? In what way?*

*Prompt:* ask: Where was the driver driving? Rural or urban road? Speed limit? Heavy or light traffic? Predictable or unpredictable traffic flow? Pedestrians? Pressure from other drivers?
